# Lymphatic and vascular anatomy define surgical principles for bowel-sparing radical treatment of ileal tumors

**DOI:** 10.1007/s00464-025-11590-y

**Published:** 2025-02-26

**Authors:** Teodor Vasic, Milena Stimec, Bojan Vladimir Stimec, Dejan Ignjatovic

**Affiliations:** 1 Clinic for Digestive Surgery, University Clinical Centre of Serbia, Faculty of Medicine, University of Belgrade, Belgrade, Serbia; 2 Anatomy Sector, Teaching Unit, Faculty of Medicine, University of Geneva, Geneva, Switzerland; 3 Department of Digestive Surgery, Akershus University Hospital, Nordbyhagen, Norway; 4 Institute of Clinical Medicine, Faculty of Medicine, University of Oslo, Oslo, Norway

**Keywords:** Ileal tumor, D3 volume, Ileal artery, Lymphatic anatomy, Operative technique

## Abstract

**Background:**

There is no consensus on the level of vascular ligation and the extent of lymphadenectomy in the treatment of ileal tumors. This study aims to define lymphovascular bundles of the terminal ileal artery (TIA) and subsequent ileal arteries. It also aims to extrapolate results from two distinct methodologies to define the level of arterial ligation and the dissection area for radical and bowel-sparing surgery.

**Methods:**

Analysis of 3D-CT mesenteric vascular reconstructions of 104 operated patients. The second dataset consisted of 5 human cadavers for anatomical dissection. In one case, harvested viscera underwent the superior mesenteric artery (SMA) perfusion after ligation of the TIA.

**Results:**

The calibers of the first three ileal arteries were: 2.67 ± 0.98 mm, 2.22 ± 0.78 mm, 2.31 ± 1.24 mm. The distances from the first three ileal arteries to the ileocolic artery (ICA) origin were: 12.45 ± 8.79 mm, 27.45 ± 13.47 mm, and 43.04 ± 16.94 mm. The SMA trifurcated in 61 (59%) of cases and bifurcated in 43 (41%). In 89 cases, the combined ICA + first jejunal artery caliber (6.7 ± 1.6 mm) was greater than the TIA caliber (4.84 ± 1.42 mm). The ileal artery lymphatic clearances were 0.85 mm to the preceding vessel. In the D3 volume at the ICA origin, 3–8 lymph nodes were observed. Internal calibers of the small bowel marginal artery, after selective TIA ligation and the SMA perfusion, were: proximal jejunal part 0.417 mm and distal ileal part 0.291 mm.

**Conclusions:**

Ileal tumors are irrigated through the TIA, which can be ligated without consequences. Lymphadenectomy should encompass the adjacent vessels (1st jejunal artery, ICA) and can include the central nodes (D3 volume) at the surgeon’s preference. Preserving the adjacent vessels and the marginal artery is paramount for bowel-sparing surgery.

**Graphical abstract:**

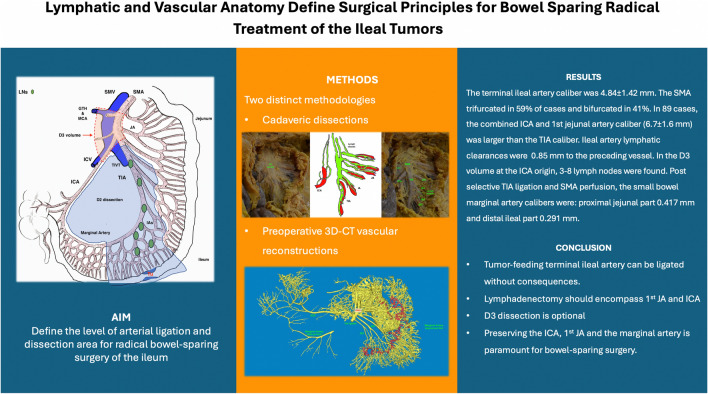

**Supplementary Information:**

The online version contains supplementary material available at 10.1007/s00464-025-11590-y.

The interest in surgical anatomy and techniques deployed in treating small bowel (SB) tumors was aroused early in the 1920s when French authors observed that SB lymphatic vessels were up to four times more diverse than the blood vessels [1]. The conclusion at the time was that the jejunum and ileum have a wide field of lymphatic drainage, making radical and bowel-sparing surgery for SB tumors difficult [2]. It is worth noting that as early as 1930, Cokkins et al. highlighted the crucial function of mesenteric arches in blood supply to the SB [3]. They discovered that ligating the superior mesenteric artery (SMA) below the middle colic artery (MCA) origin and preserving the two most cranial jejunal arteries (JAs) allowed perfusion across the areas served by the seven cranial branches of the SMA and the distal ileum. However, this resulted in a non-perfused segment linked to the four caudal branches of the SMA. Additionally, Buffachi et al. showed that blood flow to most of the SB is sufficient when only the first upper three JAs remain perfused [4]. In addition, Cokkins found that selective ligation of the terminal mesenteric arcade or recta arteries led to a distinctly non-perfused segment [3]. Most of these data have, as of yet, not been completely incorporated into the operative techniques deployed for the treatment of SB tumors.

The literature recommends that all resectable SB tumors, localized proximal to the last 20 cm of the ileum, should undergo organ-sparing segmental SB resection with lymphadenectomy while preserving the ileocolic vessels and mesenteric arcades [5]. Still, the tumor-feeding artery and the precise level of its ligature, as well as the extent of lymphadenectomy, are often vaguely defined [6–9]. Moreover, no landmark can be found that clearly depicts the transition point between the ileum and the jejunum [2]. Consequently, a single procedure, a “wedge” SB resection, is typically employed across the entire organ length, reaching an average of up to 7 m (unfortunately referred to as the jejunoileum) [10, 11].

Introducing complete mesocolic excision (CME), Central Vascular Ligation, and/or level III lymph node (LN) dissection (D3) in colorectal surgery seems to have improved survival rates in patients with advanced disease while simultaneously decreasing local recurrence rates and significantly increasing the number of harvested LNs [12–15]. The main goal of these procedures is to remove whole and uninjured arterial lymphovascular (LV) bundles and prevent lymphatic spillage during surgery [16, 17]. Nevertheless, the results of radical surgical resection of ileal tumors fail to meet the current standards of colonic cancer treatment [18]. The reason for the inferior number of harvested LNs (8 vs.12), increased local recurrence, and decreased overall survival can be related to insufficient surgical technique and tumor biology, but it can also be affiliated with the anatomy of the LV bundles and the complete lymphatic clearances of the ileal arteries (IAs) [19]. So far, the LV bundles and lymphatic clearances of the IAs have not been described in the literature.

This study aims to analyze the microanatomic specifics of ileal arterial irrigation and venous and lymphatic drainage through two distinct methodologies: anatomical dissection and 3D reconstruction of the mesenteric vascular anatomy. Furthermore, it aims to define the operative technique for treating ileal tumors proper, located at least 20 cm proximal to the ileocolic valve.

## Materials and methods

### Study settings

In this study, we utilized two distinct sets of data. The first source comprised data from a 3D-CT mesenteric vascular anatomy reconstructions dataset involving 104 patients enrolled in the “Surgery with Extended Mesenterectomy for SB Tumors” clinical trial. This trial received approval from the Regional Ethical Committee South-East, Norway, and was registered on *clinicaltrials.gov* [20]*.* Before surgery, each patient provided informed consent. The inclusion criteria were age above 18 years, radiologically or scintigraphically diagnosed SB extra-duodenal tumors, and ability to tolerate general anesthesia. As part of the trial, the mesenteric vascular anatomy of the area was reconstructed in 3D based on the pre-operative staging CT dataset for all patients. For the purpose of the study, according to Spasojevic et al. the predefined D3 area is determined by the following borders [21]:

1. Cranial border: 5 mm cranially from the line that passes through the origins of the MCA and the gastrocolic trunk of Henle (GTH);
2. Lateral border: 1 cm to the right of the line along the right side of the superior mesenteric vein;
3. Caudal border: 1 cm distal to the line that runs through the origins of the ileocolic vein (ICV) and the ileocolic artery (ICA);
4. Medial border: the line along the left side of the SMA;
5. Anterior surface: the peritoneum
6. Posterior surface: the mesofascial plane.

The second dataset consisted of 5 embalmed human bodies (two females and three males, aged 65–93 years) from the body donor program of the Anatomy Sector, Faculty of Medicine, University of Geneva, Switzerland. The use of the human cadaveric material did not require an Institutional Review Board (IRB) decision, as it was done following the Federal Act on Research involving Human Beings (Human Research Act, HRA), Guidelines of the Swiss Academy of Medical Sciences, and the principles of the Swiss Society of Anatomy, Histology, and Embryology [22–24]. Donors formally consented to the use of their body parts for research purposes after death by signing a body donation statement form [25].

### Preoperative CT reconstruction

All patients in the first dataset underwent standard preoperative investigation for SB tumors, as per Norwegian guidelines. This involved an abdominal and pelvic CT scan, which was used for 3D vascular reconstruction. No new scans were ordered. The CT data set was analyzed using a 2D multiplanar reconstruction with a maximum intensity projection and a 3D volume-rendering technique using the FDA-approved OSIRIX MD v.14.0 64-bit image-processing application software (Pixmeo, Bernex, Switzerland). The root of the mesentery and adjacent structures were precisely depicted using manual segmentation via the serial application of Region of Interest (ROI) through editing tools: Open polygon, Pencil, and Repulsor. After attributing pixel values outside ROIs to air and inside to the original value, the virtual 3D model was obtained by volume-rendering (VR). The identification, internal caliber, and course of the vascular structures within the root of the mesentery were analyzed according to the postulates for CT angiography of mesenteric vessels [26].

### 3D vascular reconstruction data collection

The 3D-reconstructed images and written reports were utilized for data collection, which involved measuring the internal calibers of the ICA, the MCA, the right colic artery (RCA), the ICV, the first jejunal artery (JA), the terminal ileal artery (TIA), and the first six IAs and ileal veins (IVs). Measurements included distances from the ICA origin and IAs origins, as well as between ICV origin and IVs origins. The IAs were counted from the ICA origin caudally, and IVs were counted caudally to the ICV origin. The caliber and distances of the first 6 IA and IVs are reported, as this number of vessels was typically identified. The first JA was identified as the most proximal artery cranial to the ICA origin, while the first IA was the most proximal artery caudal to the ICA origin, in compliance with the definition of VanDamme and Bonte [27]. The position of the ICA/RCA relative to the SMV was noted as anterior or posterior to the SMV. The position of the Terminal Ileal Venous Trunk (TIVT) relative to the TIA was noted as anterior or posterior to the TIA. The study also recorded the cases where the combined calibers of ICA/ICV and the first JA/first JV exceeded the caliber of the TIA/TIVT.

Additionally, the SMA branching pattern at the ICA’s origin was described. Bifurcation involved two branches of the SMA, namely ICA and TIA. Trifurcation consisted of three branches originating from the SMA: ICA, TIA, and first JA. The right side of the TIA was examined for arterial branches.

### Anatomical dissection

The exclusion criteria for this study were pathology and/or prior surgery of the infracolic compartment of the abdominal cavity. The bodies were perfused transarterially with a Jores solution (1875 mL of 40% formaldehyde, 750 mL of chloralhydrate, 750 mg of Carlsbad salt, and 12,375 mL of distilled water) and stored in the refrigerator at 4 °C. After the corpses were adapted to the ambient temperature, the minute dissection began accessing the right infracolic compartment of the peritoneal cavity. The transverse mesocolon was pulled cranially, and the SB mesentery was fanned out from the proximal jejunum to the terminal ileum. Following the ICA fold, the SMA and SMV were identified, and the anterior leaf of the mesentery was carefully removed from the prospective area. The next phase included microdissection, carried out by narrow spatulas, micro-dissection scissors, small tweezers, and curved forcepses, with the help of a 5× magnifying glass with a fluorescent ring lamp. The subserosal fatty tissue was removed by gentle scraping, and within it, the LV bundles and LNs were identified. These structures followed closely the arborization of the SMA branches and the SMV affluents, which were also disposed. The course of the lymph vessels was followed, and they were separated from the underlying nerve plexuses and connective tissue without disturbing their native anatomical position. The working field was regularly sprayed with a phenol solution throughout the dissection, preserving the original texture. The syntopy of the anatomical entities in the area was carefully observed and noted. The following measures were taken with the aid of the digital Vernier electronic caliper (S Cal Work 150 mm, Sylvac, Crissier, Switzerland):

- External calibers of the first JA (from the ICA origin), the first 3 IAs of the ICA, and of the SMA immediately below the ICA origin
- Distance between each of the three IAs and its neighboring accompanying lower lymph vessel
- Caliber and crossing pattern of the first IVs corresponding to the three IAs

We also noted the number of LNs associated with the ICA and ICV origins within the D3 volume [21].

### Mimics segmentation and 3-matic morphometry

In one of the 5 cases, the harvesting of viscera was performed on a fresh cadaver so that it could undergo imaging. This specimen consisted of the pancreas, duodenum, mesentery, jejunoileum, cecum, ascending colon, and transverse mesocolon, all harvested en bloc. The root of the SMA was cannulated and thoroughly rinsed with tepid water to evacuate the blood clots. Then, following the ileocecal fold, the SMA was identified and ligated immediately below the depart of the ICA. Afterward, the SMA was carefully injected with a solution consisting of 20% of barium-sulfate, 60% of liquid latex, and 20% of isotonic saline. The injection was halted upon the appearance of epi-intestinal vessels on the SB surface. The specimen underwent CT imaging, performed on a LightSpeed VCT (GE Medical Systems), with the following scanning parameters: slice thickness 0.625 mm, spacing between slices 0.300 mm, voltage 120 kV, X-ray tube current 500 mA; single collimation width 0.625 mm; scan-pitch ratio 0.984:1. The excisate was afterward submerged into the same Jores solution as mentioned above. The specimen underwent image analysis using Mimics Medical Image Processing Software ver. 24.0, and 3-matic medical software ver. 16.0, both for Windows 10 Pro × 64 (Materialise NV, Leuven, Belgium). The tools applied were Threshold, Region Grow, Multiple Slice Edit with Interpolation, Boolean Operation, Crop Mask, and Morphology Operations. After obtaining the initial synoptic mask, the paraintestinal arcade was depicted as a separate entity. The segmentation results were exported as still images, videos, STL files, and 3D PDFs.

### Statistical analysis

Parametric or nonparametric methods analyzed data. Observed characteristics were expressed as mean values, standard deviation, and median with interquartile range (IQR). We used the Kolmogorov–Smirnov test to assume normality. IBM SPSS Statistics 21 (Chicago, Illinois, USA) was used for the analysis.

## Results

### 3D vascular reconstruction dataset

A total of 104 patients were included in the first dataset (63 (60.6%) males, median age 62.5 (IQR 18) years).

#### The arteries

The morphometric measurements of the ICA, RCA, MCA, TIA, IA 1–6, and first JA are shown in Table 1. The ICA, MCA and TIA were present in all cases, while the RCA was found in 21 cases (20.2%). In one case, ICA and MCA had a common trunk. The MCA crossed SMV anteriorly in all cases. The RCA crossed the SMV anteriorly, except in one case. The ICA crossed SMV anteriorly in 35 cases (33.7%) and posteriorly in 69 cases (66.3%). The TIA crossed SMV/TIVT anteriorly in 15 cases (14.4%) and posteriorly in 89 cases (85.6%). The median number of JAs was 4 (2). The median number of IA was 6 (4), and the number of identified IAs ranged from 1 to 18. The mean distance between the first two IA (IA1-IA2) was 15.4 ± 8.8 mm. The caliber of the TIA was 4.84 ± 1.42 mm. In 89 cases, the combined ICA + first JA caliber (6.7 ± 1.6 mm) was greater than the TIA caliber, while in 15 cases, it was lower. SMA trifurcated in 61 (59%) cases, while bifurcation was present in 43 (41%).

**Table 1:** Descriptive morphometric statistics for the colic and ileal arteries/veins, derived from the 3D-CT reconstruction of the vascular anatomy

The arteries									
Variables	First JA	IA1	IA2	IA3	IA4	IA5	IA6	ICA, RCA, MCA	TIA
Caliber	3.1 ± 1.0	2.67 ± 0.98	2.22 ± 0.78	2.31 ± 1.24	2.03 ± 0.78	2.06 ± 0.61	1.94 ± 0.69	3.5 ± 0.9, 2.2 ± 0.8, 3.2 ± 0.2	4.85 ± 1.42
ICA distance	10.9 ± 5.7	12.45 ± 8.79	27.45 ± 13.47	43.04 ± 16.94	53.40 ± 18.01	65.33 ± 21.51	79.67 ± 23.59	N/A, 13.96 ± 5.8, 25.14 ± 11.2	N/A

The veins									
Variables	First JV	IV1	IV2	IV3	IV4	IV5	IV6	ICV	TIVT
Caliber	9.2 ± 5.1	5.60 ± 2.00	3.60 ± 1.26	3.14 ± 1.43	2.62 ± 0.96	2.40 ± 0.87	2.26 ± 0.71	0.47 ± 0.16	0.80 ± 0.19
ICV distance	19.5 ± 9.0	18.69 ± 10.13	40.99 ± 13.96	62.00 ± 15.71	76.45 ± 19.01	89.56 ± 19.48	103.28 ± 27.11	N/A	N/A

All metric measurements are given in millimeters

*ICA* ileocolic artery, *RCA* right colic artery, *MCA* middle colic artery, *JA* jejunal artery, *IA* (IA2, i.e. second ileal artery), *TIA* terminal ileal artery, a terminal portion of superior mesenteric artery below ICA origin, *JV* jejunal vein, *IV* ileal vein, *TIVT* terminal ileal venous trunk (terminal branch of Superior Mesenteric Vein), *ICV* ileocolic vein. *ICA distance* the distance of each IA or JA to the ICA, *ICV distance* the distance of each IV or JV to the ICV, *N/A* not applicable

#### The veins

The caliber and distances for the ICV, IV1-6, and JV1 are shown in Table 1. The median number of IVs was 4 (3), and the number of identified IVs ranged from 1 to 14. The mean distance between the first two IVs (IV1-IV2) was 22.3 ± 11.5 mm. TIVT was present in all patients and crossed SMA/TIA anteriorly in 89 (85.6%) cases. In 96 cases, the combined ICV + JV1 caliber was greater than the TIVT caliber, while in 8 cases, it was lower. SMV trifurcated in 53 (50.8%) cases, while bifurcation was present in 51 (49.2%).

### Anatomical dissection dataset

**Fig. 1:**
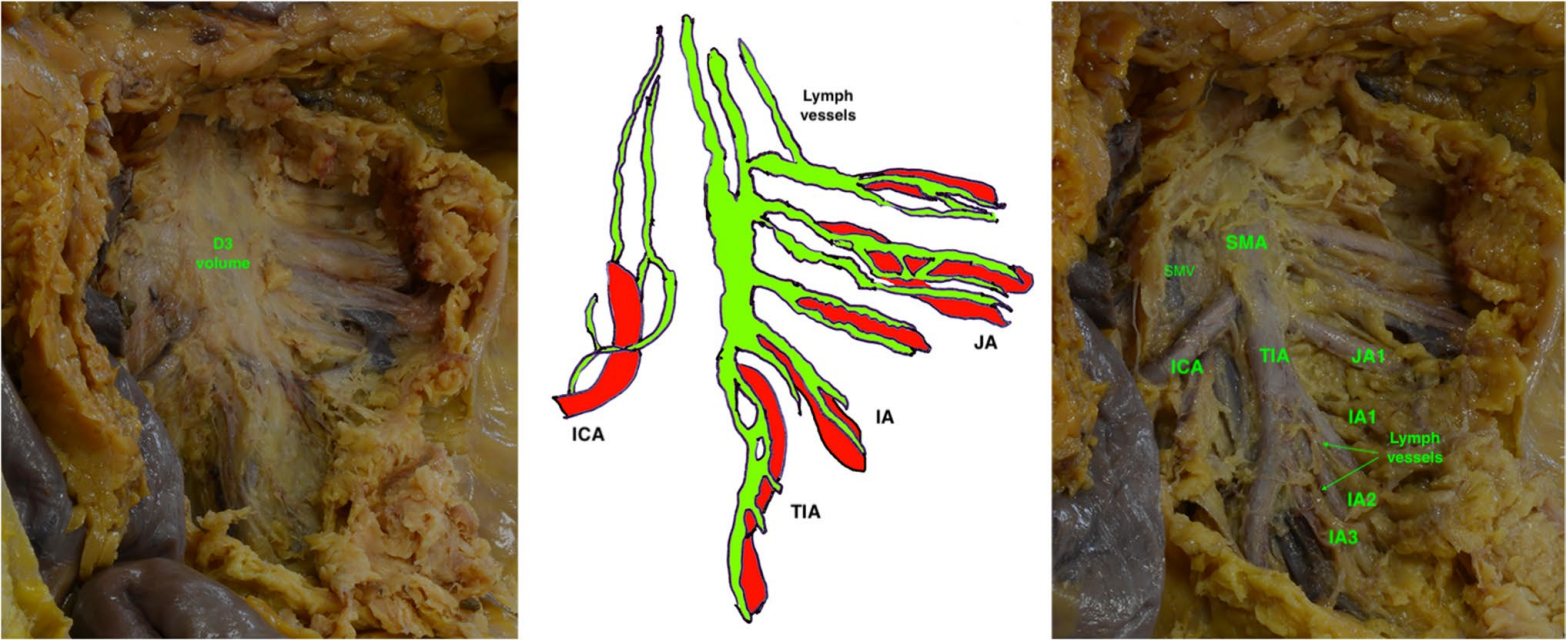
Anatomical dissection of the lymph vessels at the level of the SMA tripod. ICA ileocolic artery, JA jejunal artery, IA (IA2, i.e., second ileal artery), TIA terminal ileal artery, a terminal portion of superior mesenteric artery below ICA origin, D3 volume [21]

The results of the cadaveric dissections are presented in Table 2. The second dataset consisted of three male and two female cadavers with a mean age of 80.6 years. The external calibers of the first three IAs (counting from the ICA origin) were as follows: 3.35 mm, 2.39 mm, and 2.86 mm, respectively. The mean SMA caliber just below the ICA departure was 5.76 mm. In one case, there was a common MCA-ICA trunk, and the SMA caliber was measured below its origin. The analysis of LNs in the small portion of the D3 volume related to the ICA origin revealed between 3 and 8 nodes in this area. In the area irrigated by the mentioned 3 IAs, there were 1–2 main IVs, which mainly crossed the SMA anteriorly and had a mean caliber of 6.12 mm. The lymph vessels in the proximal ileal portion of the mesentery had a mean distance of 0.85 mm to the preceding IA they accompanied. The ileal LV network distally showed less distinction, with overlapping and mutual anastomosing of lymph vessels, making it difficult to establish the precise distance between the remainder of IAs and their lymph vessels. However, the final lymph draining ducts followed the SMA trunk cranially, forming the definite large collector lymph channel at the level of the SMA jejunal portion (Fig. 1). Parallel to it, but to the left, was a distinct LV network following the ileocolic vessels. It is important to note that the IAs emerged exclusively from the left-hand side of the SMA.

**Table 2:** Descriptive statistics for the anatomical dissections of human cadavers

Cadaver	Age	Sex	IA cal	LyNo	LyVe dist	TIA cal	ICA cal	IV cal	1st JA cal
1	65	M	3.00; 2.55; 2.20	5	0.50; 0.65; 1.05	4.7	5.25 (cTr with MCA)	5.20 ant	3.65
2	93	F	2.20; 1.25; 3.30	4	0.90; 0.70; 0.60	4.3	3.35	8.55 ant; 2.85 ant	2.9
3	70	F	3.65; 2.15; 3.10	8	1.40; 0.85; 0.75	5.6	3.95	10.00 ant; 7.20 ant	4.15
4	86	M	4.65; 3.55; 3.25	4	1.6; 0.4; 1.2	7.40	5.80	4.3 ant	6.1
5	89	M	3.20; 2.80; 2.45	3	0.90; 0.65; 0.55	6.8	4.2	5.4 post	3.70

All metric measurements are given in millimeters

*IA caliber* external diameter of IA from the ICA origin downwards, *LyNo* lymph nodes in D3 volume on the ICA origin, *TIA* terminal ileal artery external diameter, SMA portion below ICA origin, *ICA caliber* ICA external diameter, *LyVe dist.* LyVe distal to IA (from ICA), *IV caliber* external diameter of IVs from the ICV origin downwards, *1st JA caliber* 1st JA external diameter, cranial to the ICA, Multiple values separated by the semicolon represent separate blood vessel (IA, lymph vessel, IV); Abbreviations “ant” and “post” in IV caliber column represent the course/position of the IV to the adjacent IA, *cTr* common trunk

#### Mimics segmentation and 3-matic morphometry results

**Fig. 2:**
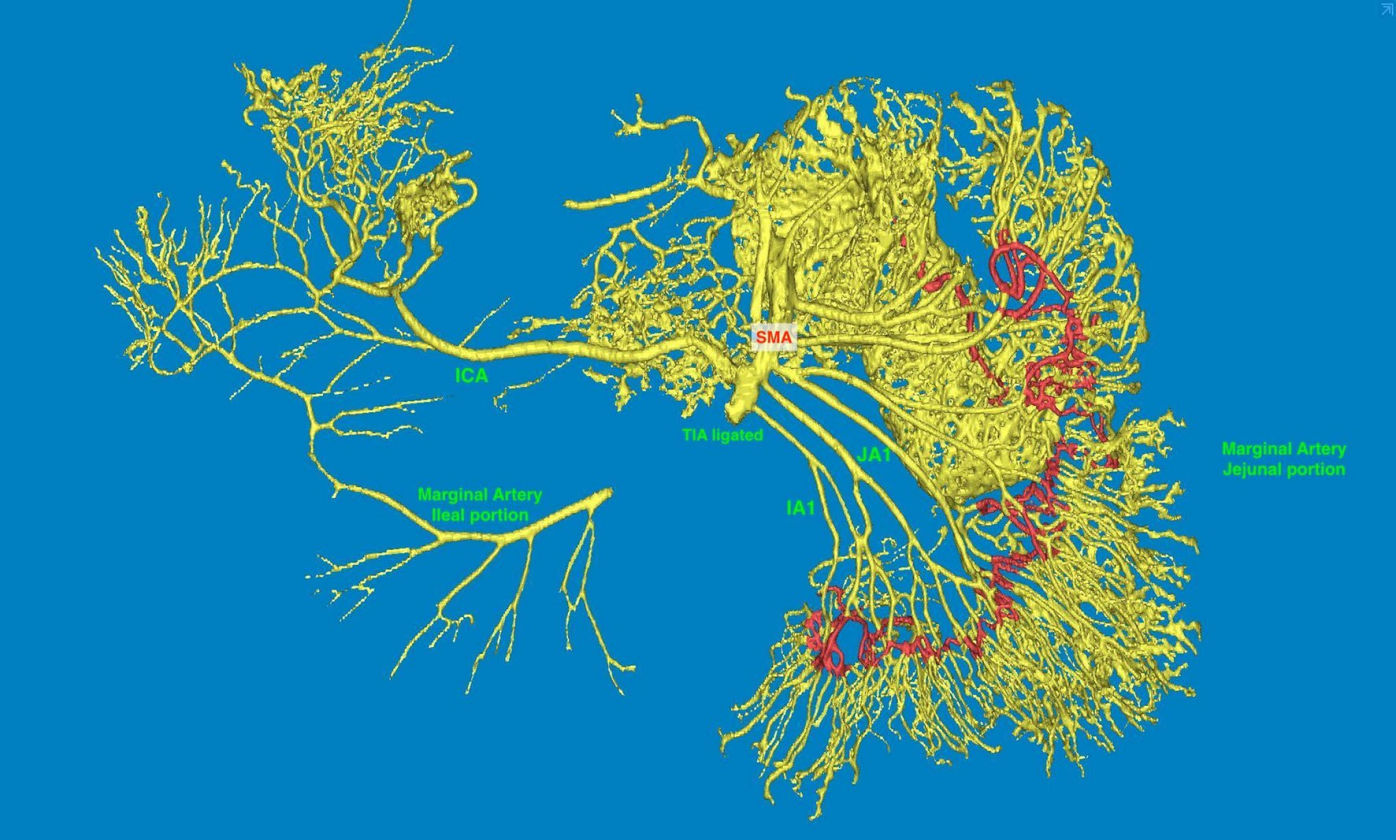
Mimics segmentation of the perfused excisate’s paraintestinal marginal artery. SMA superior mesenteric artery, ICA ileocolic artery, JA1 first jejunal artery, IA1 first ileal artery, TIA terminal ileal artery, a terminal portion of the superior mesenteric artery below the ICA origin

After thresholding, segmentation, and calculating part procedure, the 3D mask revealed that the ligature was, in fact, just below the first ileal artery (as counted from the ICA). The arterial arborization presented a retrograde filling of the ileal para-intestinal arcade via ICA and its ileal branch (Fig. 2). Considering that the highest injection pressure was at the proximal part of the SMA, one clearly visualizes better filling of the duodenal and proximal jejunal vascular tree. The following calibers of the marginal artery of the SB (i.e., the para-intestinal arcade-jejunal portion) were obtained: upper internal jejunal caliber of 0.417 mm, lower internal ileal caliber of 0.291 mm, length (over the surface) of 460.59 cm.

## Discussion

Our research highlights the importance of LV arterial bundles and their anatomy when operating on ileal tumors. The current definition of the ileum is somewhat vague, not providing the surgeon with a clear landmark between the ileum and jejunum. This missing landmark also seems to perpetuate the wedge or “pizza pie” and/or “all in one” type of SB resection [11]. This fact encourages us to explore the possibility that surgical procedure for treating proper ileal tumors could and/or should differ from those employed for the jejunum [28]. Namely, jejunal tumors can be irrigated through several distinct arteries arising from the SMA, while proper ileal tumors are considered a single-vessel disease if the definition of VanDamme and Bonte is accepted [27].

Gillot describes the SMA tripod, essentially placing the origin of the ICA between the ileal and jejunal arteries, something that is not only practical for surgeons to accept but also easy to find at surgery [29]. VanDamme and Bonte further state that the distal SMA branches fuse with the ileal branch of the ICA [27]. Our results confirm both the trifurcation/bifurcation pattern and the anastomosis to the distal marginal artery along the SB. No arterial origins were detected on the right-hand side of the TIA, indicating that preservation of the ICA and its ileal branches is paramount for bowel-sparing oncologic surgery. Numerous small caliber IAs arise from the left-hand side of the TIA, making a denser network of ileal arcades, also described in Grays’s Anatomy (8–12 IAs) [2]. Their smaller caliber (compared to JAs) renders the irrigation provided by these vessels of lesser importance when compared to the calibers of the vasa recta and the marginal SB artery (traversing the whole length of the SB mesentery), as demonstrated through our 3D injected model (Fig. 2). The joint caliber of the ICA and JA1 also contributes to this network, most often of larger caliber than of the potentially ligated TIA.

On the other hand, anatomical dissection clearly defines the LV bundles of the SMA tripod, TIA, the first JA, and the IAs (Fig. 1, Digital Supplemental Content). The LV bundle of the ICA has been previously described [21].

**Fig. 3:**
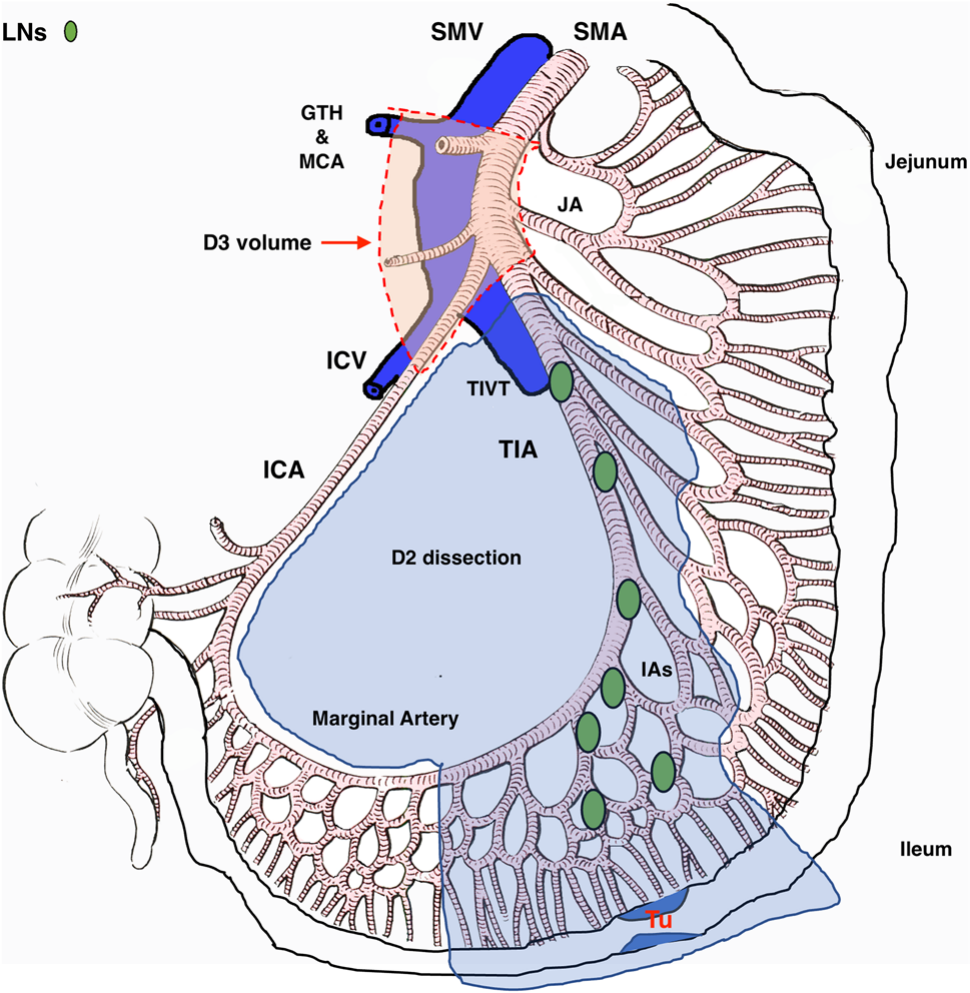
The D2 and D3 levels of dissection and the proposed technique of extended mesenterectomy. ICA ileocolic artery, JA jejunal artery, IAs ileal arteries, TIA terminal ileal artery, a terminal portion of Superior Mesenteric Artery below ICA origin, TIVT terminal ileal venous trunk (terminal branch of Superior Mesenteric Vein), ICV ileocolic vein, SMA superior mesenteric artery, SMV superior mesenteric vein, marginal artery paraintestinal ileal arcade, Tu tumor, GTH gastrocolic trunk of henle, MCA middle colic artery. Blue line—D2 dissection line- Lymphadenectomy should follow the ICA on one side and the 1st jejunal artery on the other, to the first arcade. Red dashed line—D3 volume dissection line

The implications for surgeons and the surgery seem clear when the results of the two distinct methodologies are fused. Unlike jejunal tumors, proper ileal tumors are a one-vessel disease, and the tumor’s irrigating artery is the TIA in all cases [28]. In a D2 SB resection, when a mesenteric mass is present, the TIA should be ligated at its origin, distal to the ICA’s origin. Since the vascular dissection occurs within the vascular sheath of the SMA and TIA, it is also safe to perform lymphadenectomy along the TIA to the tumor-feeding ileal vessel when a mesenteric mass is absent; in this scenario, ligature of the TIA should be done at the level of a preceding IA. Lymphadenectomy should be performed along the ICA on one side and the first JA on the other, extending to the first arcade. It is important to note that this lymphadenectomy to the first arcade allows for preserving the remaining arcades in the mesentery, thus minimizing the loss of SB length, as illustrated in Fig. 3. This lymphadenectomy will always include LNs in the SMA tripod area (3–8 LNs). Additionally, through lymphatic mapping, Wang et al. showed that when the mesenteric mass blocks lymphatic flow, the tumor’s lymph drainage shifts longitudinally along the bowel, reaching the first open lymphatic vessel and then moving radially towards the mesenteric root [30]. The operative strategy described will encompass all similar cases. It is paramount to preserve the ICA, the first JA and the marginal artery/first arcade to ensure a complication-free postoperative period [31]. If the surgeon decides to perform a D3 SB resection, the D3 volume can be removed *en bloc* with the specimen, as we have previously described [21]. The literature indicates that LN metastases frequently occur in the D3 volume when operating on SB tumors. Bartsch et al. reported a median LN harvest of 13 LNs and the occurrence of level II macroscopic LN metastases in up to 88% of patients operated with segmental SB resection (i.e. LNs from the junction of the ICA and SMA extending to the horizontal portion of the duodenum and the lower margin of the pancreatic body) [8]. Pasquer et al. noted the median of 30 LNs resected, with the highest median LN ratio of 0.14 (the number of LN metastases to the number of LNs removed) in group 3 LNs (i.e. retropancreatic LNs). They observed the presence of “skip metastases” in group 2 LNs (i.e. along the SMA) and group 3 LNs in 57% of cases [32]. These two articles depict two extended mesenterectomy volumes that are not comparable, implying the need for standardization according to anatomical landmarks with the aim of reproducibility. Another point is that the mesentery is a three-dimensional structure in which LNs are located both anterior and posterior to the SMA/SMV [21]. Due to the LN mesenteric masses and mesenteric shrinkage that can occur, the dissection in the central mesentery does raise the risk of vascular injury, subsequent bleeding, and loss of additional SB length. During the dissection in the SMA tripod area, preoperative awareness of the ICA’s course relative to the SMV (anterior in 35 cases) could lower the risk of injury and the need for ileocecal resection in proper ileal tumor surgery. Additionally, anatomical variations in the tripod area (including one case of an ICA-MCA common trunk) have been previously reported, which adds to the anatomical complexity [33]. This highlights the importance of the 3D-CT reconstructed vascular roadmap, which reduces operative time, helps prevent injuries, and offers solutions when they happen [34–36].

Furthermore, accompanying the ligation of the TIA, the question of the TIVT ligation should be addressed. The lymphovascular bundles of the mesentery follow the arteries and not the veins [16, 21]. Consequently, the TIVT ligation point does not impact D2 or D3 dissection. However, it does affect the likelihood of vein stump thrombosis if the vein is not ligated at the confluence, resulting in a longer venous stump [37, 38].

**Fig. 4:**
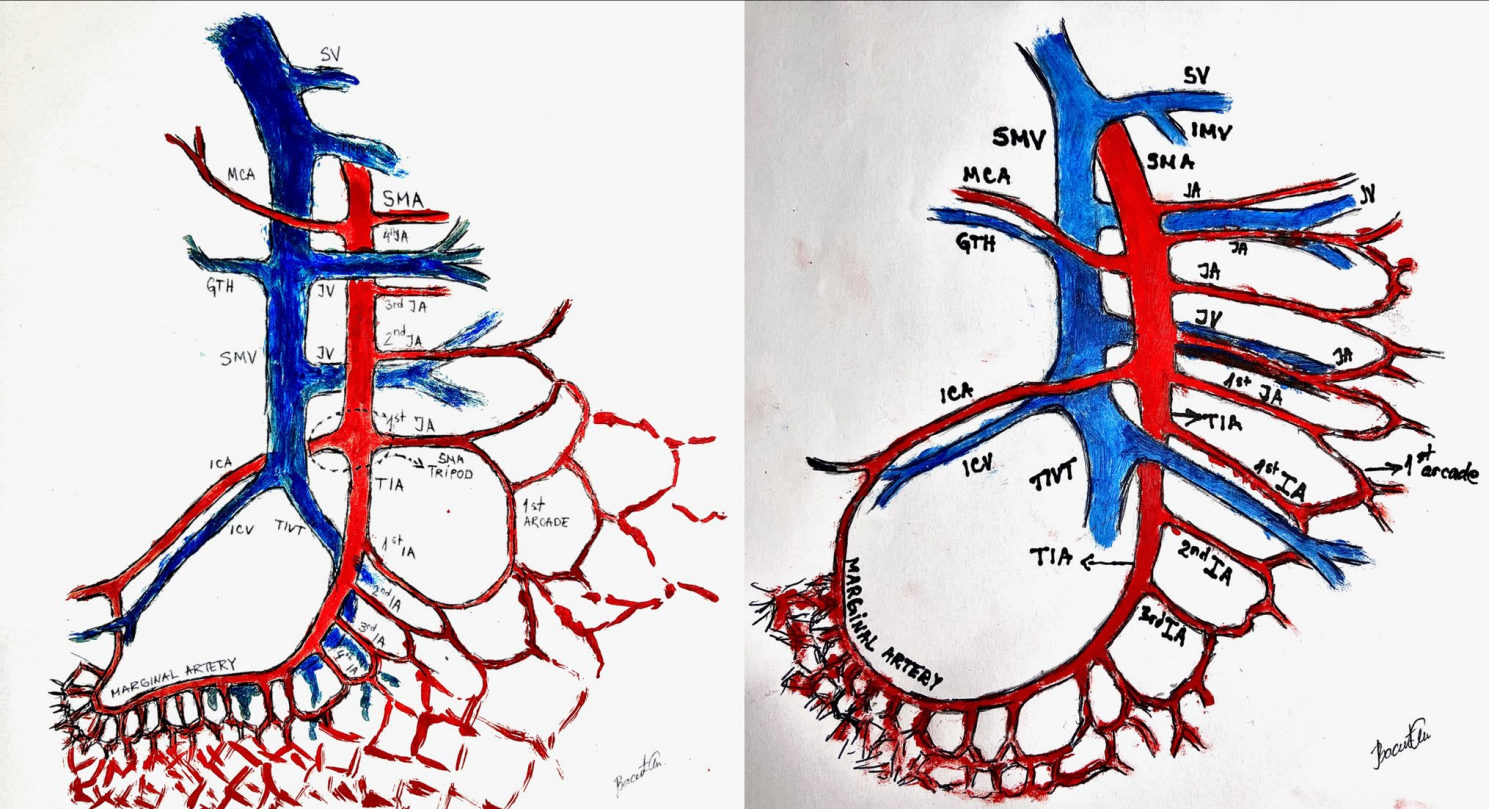
Anatomical illustration of the superior mesenteric artery and vein: branching pattern and spatial relationships. Illustration on the left: ICA posterior to the SMV, TIA anterior to the TIVT. Illustration on the right: ICA anterior to the SMV, TIA posterior to the TIVT. ICA ileocolic artery, JA jejunal artery, IA ileal artery, TIA terminal ileal artery, TIVT terminal ileal venous trunk, ICV ileocolic vein, SMA superior mesenteric artery, SMV superior mesenteric vein, marginal artery paraintestinal ileal arcade, Tu tumor, GTH gastrocolic trunk of Henle, MCA middle colic artery, JV jejunal vein, SV splenic vein, IMV inferior mesenteric vein

When all the above is considered, it paradoxically implies that a more central arterial ligation (larger caliber vessel) facilitates bowel-sparing surgery by preserving the ICA on one side and the arcades of the 1st JA on the other, preserving the vasa recta on the remaining bowel (Fig. 3). This is also clearly demonstrated through the cadaveric specimen, where the injected dye perfused the whole specimen despite the ligation of the TIA (Fig. 2). While not presenting scientific proof for this statement, it seems that the majority of patients also have an adequate sum of the ICA/JA1 calibers when compared to the caliber of the TIA. We must inform the readership that this technique is currently being performed in our clinical trial (Fig. 4).

## Conclusion

All tumors arising in the ileum are irrigated through the TIA, which can be ligated without consequences. Lymphadenectomy should encompass the proximal vessel (JA1) and the distal vessel (ICA) and can include the central nodes (D3 volume) at the surgeon’s preference. Preserving the ICA and the JA1, as well as the marginal artery, is of paramount importance for bowel-sparing surgery.

## Supplementary Information

Below is the link to the electronic supplementary material.

Supplementary file1 (MP4 180333 KB)—Digital Supplemental Content 1. The step-by-step anatomical dissection of the Terminal Ileal Artery and ileal LV bundles. SMA—superior mesenteric artery, SMV—superior mesenteric vein, TIA—terminal ileal artery, TIVT—terminal ileal venous trunk, ICA—ileocolic artery, ICV—ileocolic vein, IAs—ileal arteries, JVs—jejunal veins, LyVe—lymph vessels, D3 volume [21]

## Data Availability

Available on request.

## Declarations

### Clinical trial

“Surgery with Extended (D3) Mesenterectomy for Small Bowel Tumors” registered at https://classic.clinicaltrials.gov/ct2/show/NCT05670574.
